# Settlement characteristics and transmission of echinococcosis: a cross-sectional study in nomadic communities on the Qinghai-Tibet Plateau, China

**DOI:** 10.1186/s40249-025-01316-6

**Published:** 2025-06-12

**Authors:** Qian Wang, Yifei Wang, Zhaohui Luo, Sha Liao, Wenjie Yu, Guangjia Zhang, Liu Yang, Wei He, Zhongshuang Zhang, Diming Cai, Jun Liu, Guo Zhou, Yongzhong Li, Yan Huang, Ruirui Li, Qi Wang, Renxin Yao, Quzhen Gongsang

**Affiliations:** 1 Sichuan Center for Disease Control and Prevention, Chengdu, Sichuan China; 2 West China Hospital of Sichuan University, Chengdu, Sichuan China; 3 Tibet Center for Disease Control and Prevention, Lasa, Tibet China; 4 Sichuan Provincial People’s Hospital, Chengdu, Sichuan China

**Keywords:** Echinococcosis, Cystic echinococcosis, Alveolar echinococcosis, Settlement characteristics, Transmission, Nomadic community

## Abstract

**Background:**

Echinococcosis remains highly endemic in some nomadic communities on the Qinghai-Tibet Plateau, China, where alveolar echinococcosis (AE) and cystic echinococcosis (CE) exhibit notably high prevalence rates of 3.64% and 2.37%, respectively. Recent settlement expansion in the region has raised concerns, as smaller, remote settlements often lacked waste disposal and sewage systems, potentially facilitating echinococcosis transmission. The aim of this study is to investigate how settlement characteristics influence echinococcosis transmission.

**Findings:**

The study was conducted from 2022–2024 in nomadic communities of Shiqu County in China. The overall prevalence rate of echinococcosis in 51 settlements was found to be 2.34% (321/13,701; 95% *CI*: 2.10–2.61), which included a prevalence rate of 1.62% (222/13,701; 95% *CI*: 1.42–1.85) for AE and a prevalence rate of 0.72% (99/13,701; 95% *CI*: 0.59–0.88) for CE. The prevalence rate of AE was significantly (*χ*^2^ = 49.57, *P* < 0.01) higher than that of CE. Settlements with a smaller population size (Z = -4.27, *P* < 0.01), a greater distance to the township center (Z = 2.66, *P* < 0.01) and a higher density of owned dogs (Z = 5.90, *P* < 0.01) were associated with higher prevalence rates of CE. These associations were also observed for AE prevalence, except for the density of owned dogs.

**Conclusions:**

This study indicates that the transmission of AE was more active than that of CE in the nomadic communities. Smaller, remote settlements had higher prevalence rates for both CE and AE. The density of owned dogs was a significant risk factor for CE prevalence but not for AE prevalence. Targeted interventions are needed in these high-risk settlements. Future research should investigate how settlement characteristics interact with hygiene practices, the infection status of intermediate and definitive hosts, and their population dynamics to better understand combined effects on echinococcosis prevalence.

## Background

Echinococcosis remains a major public health concern due to its high endemicity in some nomadic communities on the Qinghai-Tibet Plateau, China, with prevalence rates reaching 3.64% for alveolar echinococcosis (AE) and 2.37% for cystic echinococcosis (CE) [1]. Both AE and CE are classified as neglected zoonotic diseases by the World Health Organization [2]. These diseases are caused by the infections with the larval stages of *Echinococcus multilocularis* and *E. granulosus *sensu lato (s.l.), respectively. Dogs are recognized as the primary definitive hosts for *E. granulosus*, while foxes and dogs are the major hosts for *E. multilocularis* [3]*.* China carries the highest burden of echinococcosis in the world [2]. A national survey conducted from 2012 to 2016 estimated that the total number of echinococcosis patients was around 166,000 in China [4], and the estimated disability adjusted life years (DALYs) associated with echinococcosis reached 322,398 [5]. A study in 2020 on the Qinghai-Tibet Plateau found that echinococcosis caused about 126,159 DALYs in local communities [6], representing 40% (126,159/322,398) of China's total echinococcosis-related disease burden. The prevalence of echinococcosis was found to be notably high on the plateau, and the disease remains endemic [7]. This part of China is characterized by a lower level of economic development compared to other parts of the country, and the region often faces challenges in terms of the availability and quality of public services[4, 6]. The economic constraints and limited access to public services were found to be associated with the higher prevalence of echinococcosis in the less-developed region [4, 6, 7].

Shiqu County was found to be highly endemic with echinococcosis, with an AE prevalence rate of 3.64% and a CE prevalence rate of 2.37% [1]. It was reported that livestock CE prevalence was 40–80%, and infection rates in dogs for *E. granulosus* and *E. multilocularis* were 8% and 12% respectively[8]. Shiqu county has been found to exhibit significant variations in echinococcosis prevalence among communities [1, 9]. The presence of a large number of definitive hosts, including dogs and foxes, and intermediate hosts, such as yaks, sheep, and small mammals, was considered to be conducive to the endemicity of CE and AE in this region [1, 3, 8, 9]. The livestock industry is a key income source for local people, and pastoral nomadism is the traditional way of life [10]. In recent years, settlement construction has become increasingly popular as a means to improve the livelihoods of the nomadic pastoralists in the region [11]. A study found that less populated and remote settlements on the Qinghai-Tibet Plateau may lack adequate domestic waste disposal systems and have limited access to public services [12]. Field visits suggest that settlement characteristics such as area, population size, number of owned dogs, and distance to the township center (where public service facilities are located) may influence sanitation practices, hygiene conditions, and the accessibility of public services. These factors could potentially exacerbate environmental contamination by *Echinococcus* spp. eggs spread by canines. As echinococcosis in humans results from accidental ingestion of eggs shed by infected definitive hosts (e.g., dogs, foxes) [3]. These settlement characteristics could act as risk factors for the transmission of echinococcosis. Therefore, the objective of the study is to examine the above assumption in the nomadic communities on the Qinghai-Tibet Plateau.

## Methods

### Study area

The 51 study settlements were selected from Shiqu County, Garzê Tibetan Autonomous Prefecture, Sichuan Province, China—a region with well-documented high human echinococcosis prevalence [1, 6]. Situated on the eastern Qinghai-Tibet Plateau (32° 19′ 28″ N–34° 20′ 40″ N, 97° 20′ 00″ E–99° 15′ 28″ E), the county borders Qinghai Province to the east, north, and west, and the Xizang Autonomous Region to the south. With a population of 106,000 (98% Tibetan ethnicity), the county sustains livelihoods primarily through livestock husbandry. The county spans 25,191 km^2^ (19,000 km^2^ grazing land) at an average altitude of 4200 m above the mean sea level, characterized by a cold semi-arid climate (mean annual temperature: − 1.6 °C; precipitation: 596 mm) [10]. The study obtained ethical approval from the Ethical Committee of the Sichuan Center for Disease Control and Prevention.

### Data collection

The study was conducted from 2022 to 2024 in nomadic communities of Shiqu County in China. Data on settlement characteristics such as area, population size, number of households, and number of owned dogs were collected from village commissions and Shiqu County Center for Disease Control and Prevention (Shiqu CDC) by reviewing archival records and files. Patient data were provided by the Shiqu CDC, with all cases confirmed as locally acquired infections. Aowei digital mapping system (Beijing Yuanshenghuawang Software Co., Ltd.) was utilized to measure settlement characteristics, such as settlement area and the distance from settlement to township center where public institutions like healthcare and veterinary institutions are located. After obtaining informed consent, data on age, gender, and residence location were gathered through structured interviews with patients or their guardians, conducted with the assistance of local village doctors. The Aowei digital mapping system was utilized to document patients' geographic locations of residences during the data collection process.

### Data management and analysis

Data for each settlement, including the number of CE and AE patients, settlement area, population size, number of owned dogs, number of households, and distance to the township center, were recorded in a WPS Spreadsheet (Kingsoft Co., Ltd., Beijing). Additional detailed information, such as patients' age, sex, geographic location, and whether they had been diagnosed with AE or CE, was entered into a separate WPS Spreadsheet. Statistical analysis was performed using the statistical software R 4.3.3 (Lucent Technologies, Jasmine Mountain, USA). Descriptive statistics, including percentages, mean ± SD, median, and 95% confidence intervals (*CI*s), were calculated to summarize settlement characteristics and the demographics of patients. The *t*-test and chi-square test were employed to compare means and prevalence rates, respectively. A linear regression model was applied to examine the relationships between settlement area and number of households, population size, and density of owned dogs, following logarithmic transformation of the data. For both univariable and multivariable analyses, a generalized linear model (GLM) with a Poisson distribution was used to identify statistically significant risk factors influencing the prevalence of CE and AE; the population size of each settlement was used as an offset in the model to adjust for the impact of population size differences between different settlements on the dependent variable. In the multivariable analysis, a backward stepwise approach was employed to optimize the model, with the Akaike information criterion (AIC) minimized to achieve the best fit. A *P-*value less than 0.05 was considered significant for all statistical tests.

## Results

### General information

This cross-sectional study investigated 51 settlements comprising a total population of 13,701 individuals during 2022 to 2024. Epidemiological analysis showed a significant difference in prevalence between the two echinococcosis: AE (1.62%, 222 cases; 95% *CI*: 1.42–1.85%) was more common than CE (0.72%, 99 cases; 95% *CI*: 0.59–0.88%), with statistical significance (*χ*^2^ = 49.57, *P* < 0.01). The mean age of AE patients (mean ± SD: 42.63 ± 2.25 years, *n* = 222) was significantly lower than that of CE patients (47.55 ± 2.84 years, *n* = 99; *t* = 6.181, *P* < 0.05). With respect to gender, 39.19% of AE patients and 38.38% of CE patients were male, while 60.81% and 61.62% were female, respectively. Chi-square tests revealed no significant gender-based differences between the two patient groups (all *P* > 0.05).

Settlement characteristics exhibited considerable heterogeneity across multiple parameters; therefore, the median was employed as a measure of central tendency: population size, 20 individuals; settlement area, 24,353 m^2^; distance to township center, 11.83 km; number of households, 5; density of owned dogs, 190.64 dogs/km^2^.

Associations between settlement area and number of households, population size, and number of owned dogs per 1 km.^2^

**Fig. 1:**
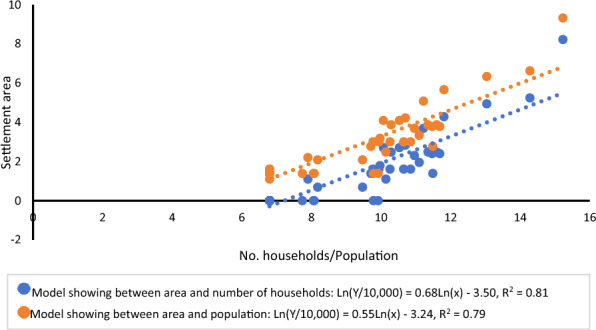
Positive associations between settlement area and number of households/population in nomadic communities of Shiqu County in China from 2022 to 2024

**Fig. 2:**
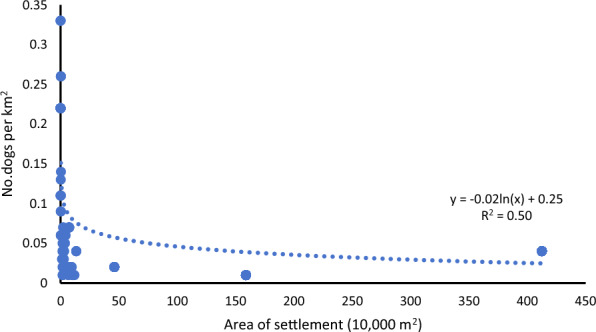
Negative association between settlement area and density of owned dogs in nomadic communities of Shiqu County in China from 2022 to 2024

Regression analysis revealed statistically significant relationships between settlement area and the number of households (Coefficient = 0.68, *t* = 18.29, *P* < 0.01) as well as settlement area and population size (Coefficient = 0.55, *t* = 19.43, *P* < 0.01), with *R*^*2*^ values of 0.81 and 0.79, respectively (Fig. 1). In contrast, settlement area showed a significant negative association with density of owned dogs (Coefficient = -0.02,* t* = -5.33, *P* < 0.01, *R*^*2*^ = 0.41) (Fig. 2).

### Associations between settlement characteristics and CE prevalence

Settlement characteristics, including area, population size, number of households, distance to the township center, and density of owned dogs, were analyzed using a Poisson regression model to assess their associations with CE prevalence. Univariable analyses revealed significant associations between CE prevalence and area (coefficient = − 0.24,* Z* = − 4.11, *P* < 0.01), population size (Coefficient = − 0.01, *Z* = − 4.27, *P* < 0.01), number of households (coefficient = − 0.00, *Z* = − 4.26, *P* < 0.01), distance to the township center (coefficient = 0.03,* Z* = 2.50, *P* < 0.01), and the density of owned dogs (coefficient = 0.00,* Z* = 6.29, *P* < 0.01) (Table 1). Multivaribale analyses further identified population size (coefficient = − 0.01, *Z* = − 4.27, *P* < 0.01), distance to the township center (coefficient = 0.09, *Z* = 2.66, *P* < 0.05), and the density of owned dogs (coefficient = 0.00, *Z* = 5.90, *P* < 0.01) as significant factors associated with CE prevalence (Table 2).

**Table 1:** Analysis of risk factors for alveolar echinococcosis (AE) and cystic echinococcosis (CE) prevalence using univariable Poisson regression

Risk factors (unit)	CE			AE		
	Coefficient	Z	*P*	Coefficient	Z	*P*
Area (km^2^)	− 0.24	− 4.11	< 0.01	− 0.30	− 8.09	< 0.01
Population	− 0.01	− 4.27	< 0.01	− 0.00	− 8.82	< 0.01
Number of households (10 households)	− 0.00	− 4.26	< 0.01	− 0.00	− 8.83	< 0.01
Distance to the township center (km)	0.03	2.50	< 0.01	0.04	6.90	< 0.01
Density of owned dogs (km^2^)	0.00	6.29	< 0.01	− 0.00	− 1.30	0.19

**Table 2:** Analysis of risk factors for alveolar echinococcosis (AE) and cystic echinococcosis (CE) prevalence using multivaribale Poisson regression

Risk factors (Unit)	CE			AE		
	Coefficient	Z	*P*	Coefficient	Z	*P*
Population	− 0.01	− 4.27	< 0.01	− 0.00	− 8.94	< 0.01
Distance to the township center (km)	0.09	2.66	< 0.01	0.05	2.71	< 0.01
Density of owned dogs (km^2^)	0.01	5.90	< 0.01	–	–	–
AIC	100.58			140.86		

“–” indicating the variable “Density of owned dogs” being excluded from the model, therefore no parameter is available. *AIC* Akaike information criterion

### Associations between settlement characteristics and AE prevalence

The associations between settlement characteristics, including area, population size, number of households, distance to the township center, and density of owned dogs and AE prevalence were evaluated using Poisson regression model. Univariable analyses revealed significant associations between AE prevalence and area (coefficient = − 0.30, Z = − 8.09,* P* < 0.01), population size (coefficient = − 0.00, Z = − 8.82, *P* < 0.01), number of households (coefficient = − 0.00, Z = − 8.83,* P* < 0.01), and distance to the township center (coefficient = 0.04, Z = 6.90, *P* < 0.01) (Table 1). Multivaribale analyses further identified population size (coefficient = − 0.00, Z = − 8.94, *P* < 0.01) and distance to the township center (coefficient = 0.05, Z = 2.71, *P* < 0.01) as significant factors associated with AE prevalence (Table 2).

## Discussion

For decades, researchers have been actively investigating the factors influencing the variation in the prevalence of echinococcosis [3, 7, 8, 13, 14]. Extensive studies have identified significant risk factors contributing to this heterogeneity, including humidity [15, 16], altitude [16, 17], livestock farming (e.g., yaks and sheep). and dog ownership [17–19], annual average precipitation [17, 20], and land use patterns [16, 20–22]. Additionally, access to public services, such as dog management programs [22, 23], safe water supplies, sanitation/hygiene facilities [24], and health education initiatives [25], has been shown to influence echinococcosis prevalence. To our knowledge, this is the first study to explore the associations between settlement characteristics and echinococcosis prevalence, offering new insights into the risk factors for the endemicity of AE and CE in nomadic pastoralist communities on the Qinghai-Tibet Plateau.

A previous study found that sparsely populated and remote settlements on the Qinghai-Tibet Plateau may struggle with inefficient domestic waste management and limited public services [12]. Such conditions can result in poor hygiene and insufficient access to essential services like dog control and livestock slaughtering inspections, which may increase the risk of echinococcosis transmission. Considering these factors, we carried out a study to examine the characteristics of settlements and their potential links to the prevalence of echinococcosis in a region where both AE and CE are highly endemic. Our results indicated that settlement area was positively correlated with the total number of households and population (Fig. 1). Conversely, a negative correlation was found between settlement area and the density of owned dogs (Fig. 2). Univariable analysis revealed that smaller settlement area, smaller population size, fewer households, greater distance to the township center, and a higher density of owned dogs were associated with a higher prevalence of CE. Similar associations were found for AE prevalence, except for the factor of density of owned dogs (Table 1). Furthermore, multivariable analysis showed that smaller population, greater distance to the township center, and a higher density of owned dogs were associated with CE prevalence. These associations also held for AE prevalence, except for the factor of the density of owned dogs (Table 2). Therefore, it appears that settlements with smaller populations and greater distances from the township center had higher prevalence rates for both AE and CE, and the risk factors for AE and CE prevalence differed.

Nomadic pastoralists on the Qinghai-Tibet Plateau have long practiced seasonal migration, settling in warmer and lower-altitude areas during winter to allow their livestock to graze on pastures preserved for winter use [26, 27]. Since 2001, a government initiative has aimed to improve living conditions by constructing permanent settlements for the nomadic communities [28]. However, smaller and more remote settlements often lack access to essential public services, such as household waste disposal and domestic sewage treatment [12]. Previous research suggested that public services, including dog management, safe water supply, and sanitation, are significant factors influencing echinococcosis prevalence [18, 22–24]. Therefore, these settlement-related limitations may increase the risk of the transmission of echinococcosis. Our study supports the hypothesis that smaller, less populated settlements may face a higher risk of echinococcosis transmission. Specifically, pastoralists in smaller settlements tend to have higher density of owned dogs (Fig. 2), likely because of limited access to public security services. The increased reliance on owned dogs for guarding livestock and property results in a higher density of these definitive hosts, which in turn increased the environmental contamination with *E. granulosus* eggs, thereby promoting the transmission of CE. Interestingly, the increased density of owned dogs did not significantly elevate the prevalence of AE. Initially, we hypothesized that larger settlements—equipped with better public services such as efficient dog management and waste disposal systems—would experience reduced environmental contamination from owned dogs compared to smaller settlements, thereby diminishing* E. multilocularis* transmission by domestic canines. While our research confirmed that *E. multilocularis* transmission to humans is indeed more prevalent in smaller settlements, this increased risk was not found to be linked to higher densities of owned dogs in these settlements. The findings suggest that residents of smaller settlements in the vast grassland may be more exposed to fox-mediated parasite transmission. Foxes, which serve as key definitive hosts in the sylvatic cycle of *E. multilocularis 3*, exhibit distinct habitat preferences: prior studies demonstrate that they favor low-density human settlements, where competition with domestic dogs and human disturbance are minimal, while avoiding high-density areas [29]. Thus, the elevated transmission observed in smaller settlements likely reflects the convergence of optimal ecological conditions for foxes—sparse human activity, limited total dog populations, and accessible resources. Our research indicated that the overall prevalence rate of AE was significantly higher than that of CE, confirming that AE is more actively transmitted within nomadic communities [8]. Young patients are considered an important marker of ongoing transmission of echinococcosis [30]. We found that AE patients were significantly younger than CE patients, suggesting a higher proportion of younger individuals in the AE group, which provides another layer of evidence to support that AE is more actively spreading in these communities.

The research was confined to some nomadic communities of Shiqu County on the Qinghai-Tibet Plateau, which might limit the broader applicability of the findings, as other regions with echinococcosis may have different environmental and cultural contexts. While settlement-level factors were explored, more in-depth investigation of individual-level behaviors and environmental micro-factors could further enhance the understanding of disease prevalence. Future studies could expand the scope and incorporate additional variables for a more comprehensive analysis.

## Conclusions

This study suggests that settlement characteristics may influence echinococcosis transmission dynamics among Tibetan nomadic communities, with smaller, remote settlements potentially associated with higher CE and AE prevalence due to limited public services. Higher owned dog density in smaller settlements was significantly associated with CE prevalence but not AE prevalence, suggesting that wild canines favoring low-density human settlements may drive greater transmission in these areas. These findings imply that tailored interventions—integrating environmental management with owned dogs and wild canines control in smaller settlements—could mitigate disease risks. While the geographical scope constrains broader generalizations, this work proposes a framework for future research on how settlement characteristics interact with hygiene practices, the infection status of intermediate and definitive hosts, and their population dynamics to better understand combined effects on echinococcosis prevalence and refine context-specific solutions.

## Data Availability

Data is available at the Sichuan Provincial Center for Disease Control and Prevention and fully accessible to all co-authors. Data can be shared with other institutions and researchers upon request.

## Abbreviations

AE: Alveolar echinococcosis
AIC: Akaike information criterion
CE: Cystic echinococcosis
CDC: Center for Disease Control
*CI*: Confidence interval
DALYs: Disability adjusted life years
GLM: General linear model
GPS: Precise geographic positioning system
WHO: World Health Organization
SD: Standard deviation

## References

[1] Yu WJ, Wang Q, Liao S, Zhang GJ, He W, Yang L, et al. Investigation on the current situation of echinococcosis in Shiqu County, Sichuan Province in 2017. J Prev Med Inf. 2018;34(05):545–9 (in Chinese).

[2] Jesudason T. Global progress report on neglected tropical diseases. Lancet Infect Dis. 2024;24(7): e420. 10.1016/S1473-3099(24)00365-7. 10.1016/S1473-3099(24)00365-7 38908383

[3] Craig PS, Hegglin D, Lightowlers MW, Torgerson PR, Wang Q. Echinococcosis: control and prevention. Adv Parasitol. 2017;96:55–158. 10.1016/bs.apar.2016.09.002. 28212791 10.1016/bs.apar.2016.09.002

[4] Wu WP, Wang H, Wang Q, Zhou XN, Wang LY, Zheng CJ, et al. Analysis of the sampling survey on echinococcosis in China from 2012 to 2016. Chin J Parasitol Parasit Dis. 2018;36(1):1–14 (in Chinese).

[5] Zhang MY, Han S, Xue CZ, Wu WT, Wu WP, Guan YY, et al. Analysis on disease of burden of hydatid disease in China. Chin J Parasitol Parasit Dis. 2018;36(1):15–20 (in Chinese).

[6] Wang Q, Yang L, Wang Y, Zhang GJ, Zhong B, Wu WP, et al. Disease burden of echinococcosis in Tibetan communities-A significant public health issue in an underdeveloped region of western China. Acta Trop. 2020;203:105283. 10.1016/j.actatropica.2019.105283. 10.1016/j.actatropica.2019.105283 31811863

[7] Han S, Kui Y, Xue CZ, Zhang YL, Zhang BG, Li QY, et al. Analysis of the endemic status of echinococcosis in china from 2004 to 2020. Chin J Parasitol Parasit Dis. 2022;40(4):475–80 (in Chinese).

[8] Wang Q, Zhong B, Yu WJ, Zhang GJ, Budke CM, Liao S, et al. Assessment of a 10-year dog deworming programme on the transmission of *Echinococcus multilocularis* in Tibetan communities in Sichuan Province. Chin Int J Parasitol. 2021;51(2–3):159–66. 10.1016/j.ijpara.2020.08.010. 10.1016/j.ijpara.2020.08.010 33220298

[9] He W, Wang LY, Yu WJ, Zhang GJ, Zhong B, Liao S, et al. Prevalence and spatial distribution patterns of human echinococcosis at the township level in Sichuan Province, China. Infect Dis Poverty. 2021Jun 5;10(1):82. 10.1186/s40249-021-00862-z. 10.1186/s40249-021-00862-z PMC8180058 34090538

[10] SCG(Shiqu County Government). Brief introduction of Shiqu county. http://www.shiqu.gov.cn/zjsq. Access on Dec 1, 2024. (in Chinese)

[11] Wang L, Wu R, Cui QW. Key Challenges in the transition from settlement to secure and stable living for herders in Tibetan areas of Sichuan Province. J Southwest Minzu Univ (Human Soc Sci Edn). 2011;32(11):114–8 (in Chinese).

[12] Zhou K, Zhang J, Song JP, Fan J. Disequilibrium and its causes on the environmental basic public services in the Qinghai-Tibet Plateau ecological barrier zone: An empirical analysis of the villages in Qinghai Province. Acta Ecol Sin. 2023;43(10):4010–23 (in Chinese).

[13] Danson FM, Graham AJ, Pleydell DR, Campos-Ponce M, Giraudoux P, Craig PS. Multi-scale spatial analysis of human alveolar echinococcosis risk in China. Parasitology. 2003;127(Suppl):S133–41. 15027610

[14] Giraudoux P, Zhao Y, Afonso E, Yan H, Knapp J, Rogan MT, et al. Long-term retrospective assessment of a transmission hotspot for human alveolar echinococcosis in mid-west China. PLoS Negl Trop Dis. 2019;13(8): e0007701. 10.1371/journal.pntd.0007701. 10.1371/journal.pntd.0007701 PMC6742415 31469833

[15] Lawson JR, Gemmell MA. Hydatidosis and cysticercosis: the dynamics of transmission. Adv Parasitol. 1983;22:261–308. 10.1016/s0065-308x(08)60464-9. 6364736 10.1016/s0065-308x(08)60464-9

[16] Yin J, Wu X, Li C, Han J, Xiang H. The impact of environmental factors on human echinococcosis epidemics: spatial modelling and risk prediction. Parasit Vectors. 2022;15(1):47. 10.1186/s13071-022-05169-y. 10.1186/s13071-022-05169-y PMC8822772 35130957

[17] Ma T, Jiang D, Hao M, Fan P, Zhang S, Quzhen G, et al. Geographical Detector-based influence factors analysis for Echinococcosis prevalence in Tibet, China. PLoS Negl Trop Dis. 2021Jul 12;15(7): e0009547. 10.1371/journal.pntd.0009547. 10.1371/journal.pntd.0009547 PMC8297938 34252103

[18] Wang Q, Qiu JM, Schantz P, He JG, Ito A, Liu FJ. Investigation of risk factors for development of human hydatidosis among households raising livestock in Tibetan areas of western Sichuan province. Chin J Parasitol Parasit Dis. 2001;19(2):93–6. 12571995

[19] Gajardo JI, Castillo MJ. Risk factors for hydatid disease in high school students in the district of Punitaqui. Chile Rev Chilena Infectol. 2017;34(3):227–34. 10.4067/S0716-10182017000300004. (in Spanish). 10.4067/S0716-10182017000300004 28991318

[20] Fischer I, Graeter T, Kratzer W, Stark K, Schlingeloff P, Schmidberger J, et al. Distribution of alveolar echinococcosis according to environmental and geographical factors in Germany, 1992–2018. Acta Trop. 2020;212: 105654. 10.1016/j.actatropica.2020.105654. 10.1016/j.actatropica.2020.105654 32783956

[21] Pleydell DR, Yang YR, Danson FM, Raoul F, Craig PS, McManus DP, et al. Landscape composition and spatial prediction of alveolar echinococcosis in southern Ningxia, China. PLoS Negl Trop Dis. 2008;2(9):e287. 10.1371/journal.pntd.0000287. 10.1371/journal.pntd.0000287 PMC2565701 18846237

[22] Craig PS, Giraudoux P, Wang ZH, Wang Q. Echinococcosis transmission on the Tibetan Plateau. Adv Parasitol. 2019;104:165–246. 10.1016/bs.apar.2019.03.001. 31030769 10.1016/bs.apar.2019.03.001

[23] Wang Q, Huang Y, Huang L, Yu W, He W, Zhong B, et al. Review of risk factors for human echinococcosis prevalence on the Qinghai-Tibet Plateau, China: a prospective for control options. Infect Dis Poverty. 2014;3(1):3. 10.1186/2049-9957-3-3. 10.1186/2049-9957-3-3 PMC3910240 24475907

[24] Barnes AN, Davaasuren A, Baasandagva U, Gray GC. A systematic review of zoonotic enteric parasitic diseases among nomadic and pastoral people. PLoS ONE. 2017;12(11): e0188809. 10.1371/journal.pone.0188809. 29190664 10.1371/journal.pone.0188809 PMC5708844

[25] Zhao J, Dawa Y, A K, Dejicuo, Lengbao, Li Z, et al. Association between echinococcosis-specific health literacy and behavioural intention to prevent echinococcosis among herdsmen on the Tibet Plateau in China: a cross-sectional study. BMC Infect Dis. 2021;21(1):101. 10.1186/s12879-021-05775-8. 10.1186/s12879-021-05775-8 PMC7821523 33482746

[26] Xu X. Study on the distribution and infection status of intermediate hosts of *Echinococcus multilocularis* in areas with high prevalence of hydatid disease. Master thesis. Chinese center for disease control and prevention. 2008. (in Chinese)

[27] Lang WW, Zhang P. Population and family characteristics of village in northern Tibetan pastoral areas and their fertility intentions: a case study of Dacun and Zongcun in Nagqu County. Tibet Studies. 2012;04:64–78 (in Chinese).

[28] Zhang J, Cui X, Wang Y, Gongbuzeren, Zhuang M, Ji B. Ecological consequence of nomad settlement policy in the pasture area of Qinghai-Tibetan Plateau: From plant and soil perspectives. J Environ Manage. 2020;260:110114. 10.1016/j.jenvman.2020.110114. 10.1016/j.jenvman.2020.110114 31941636

[29] Díaz-Ruiz F, Caro J, Delibes-Mateos M, Arroyo B, Ferreras P. Drivers of red fox (Vulpes vulpes) daily activity: prey availability, human disturbance or habitat structure? J Zool. 2016;298(2):128-138. 10.1111/jzo.12294

[30] Torgerson PR, Shaikenov BS, Baitursinov KK, Abdybekova AM. The emerging epidemic of echinococcosis in Kazakhstan. Trans R Soc Trop Med Hyg. 2002;96(2):124–8. 10.1016/s0035-9203(02)90276-2. 10.1016/s0035-9203(02)90276-2 12055797

